# Affective Fluctuations in Depressive and Anxiety Symptomatology Predict Cognitive Performance Decline in Older Adults

**DOI:** 10.1002/alz70857_101303

**Published:** 2025-12-25

**Authors:** Michael H. Malek‐Ahmadi, Angela Kuramoto, Farah Shaikh, Carla Jooste, Hamzah Al‐Jubouriy, Kathy O'Connor, David W. Coon, Alireza Atri

**Affiliations:** ^1^ Banner Alzheimer's Institute, Phoenix, AZ, USA; ^2^ University of Arizona College of Medicine‐Phoenix, Phoenix, AZ, USA; ^3^ Banner Sun Health Research Institute, Sun City, AZ, USA; ^4^ Arizona State University, Phoenix, AZ, USA; ^5^ Brigham and Women's Hospital and Harvard Medical School, Boston, MA, USA

## Abstract

**Background:**

Previous research supports that affective symptoms related to depression and anxiety are associated with lower cognitive performance in cognitively unimpaired (CU) older adults. Most studies reporting associations are cross‐sectional and have not investigated whether or how affective variability may correlate with cognitive variability or change over time. This study aimed to assess whether intraindividual variability of self‐reported affective symptoms is associated with cognitive decline.

**Method:**

Longitudinal data from 817 predominantly highly‐educated, CU middle‐age and older adults (age range 53‐102 years) were included. Cognition was measured using the Montreal Cognitive Assessment (MoCA). Center for Epidemiologic Studies Depression 10‐Item was used to assess depressive symptoms, the Penn State Worry Questionnaire was used to assess anxiety symptoms. Intrasubject standard deviation (ISD) was used to quantify year‐to‐year variability of the affective measures.

**Result:**

In primary analyses using independent mixed‐effects models adjusted for age, education, and sex, greater variability in depression and anxiety symptomatology was associated with cognitive decline. In exploratory analyses using combined models to assess for additive versus additive and synergistic effects only depressive symptom variability was significantly associated with cognitive decline. The interaction of depressive and anxiety symptom variability was not a significant predictor of cognitive decline.

**Conclusion:**

In cognitively unimpaired older adults within‐subject affective variability is associated with linear decline on cognitive performance over several years. Understanding and accounting for the potential impact of affective fluctuations on cognitive performance may improve trajectory and power estimates in observational and interventional studies, and suggest strategies to mitigate components of cognitive decline.

